# Identification of salivary mucin MUC7 binding proteins from *Streptococcus gordonii*

**DOI:** 10.1186/1471-2180-9-163

**Published:** 2009-08-11

**Authors:** Mehmet Kesimer, Nedret Kiliç, Ravi Mehrotra, David J Thornton, John K Sheehan

**Affiliations:** 1 Department of Biochemistry, Faculty of Medicine, University of Gazi, 06510 Besevler, Ankara, Turkey; 2 Department of Biochemistry and Biophysics, University of North Carolina at Chapel Hill, North Carolina 27599, USA; 3 Wellcome Trust Centre for Cell-Matrix Research, Faculty of Life Sciences, University of Manchester, Manchester, M13 9PT, UK

## Abstract

**Background:**

The salivary mucin MUC7 (previously known as MG2) can adhere to various strains of streptococci that are primary colonizers and predominant microorganisms of the oral cavity. Although there is a growing interest in interaction between oral pathogens and salivary mucins, studies reporting the specific binding sites on the bacteria are rather limited. Identification and characterization of the specific interacting proteins on the bacterial cell surface, termed adhesins, are crucial to further understand host-pathogen interactions.

**Results:**

We demonstrate here, using purified MUC7 to overlay blots of SDS-extracts of *Streptococcus gordonii *cell surface proteins, 4 MUC7-binding bands, with apparent molecular masses of 62, 78, 84 and 133 kDa from the *Streptococcus gordonii *strain, PK488. Putative adhesins were identified by in-gel digestion and subsequent nanoLC-tandem mass spectrometry analysis of resultant peptides. The 62 kDa and 84 kDa bands were identified as elongation factor (EF) Tu and EF-G respectively. The 78 kDa band was a *hppA *gene product; the 74 kDa oligopeptide-binding lipoprotein. The 133 kDa band contained two proteins; alpha enolase and DNA-directed RNA polymerase, beta' subunit. Some of these proteins, for example alpha enolase are expected to be intracellular, however, flow cytometric analysis confirmed its location on the bacterial surface.

**Conclusion:**

Our data demonstrated that *S. gordonii *expressed a number of putative MUC7 recognizing proteins and these contribute to MUC7 mucin binding of this streptococcal strain.

## Background

Saliva lubricates the oral cavity and contains innate defense related proteins (i.e. cystatins, lysozyme, proline-rich proteins, histatins, lactoperoxidase, lactotransferrin, Poly Ig receptor, DMBT1 and mucins [1,2]) that protect the surfaces of the mouth exposed to the external environment. Mucins are the major macromolecular component of the secretion and human saliva has been shown to contain at least two structurally and functionally distinct populations of mucins: the high molecular weight (M_r _> 10^6 ^Da) polymeric, gel-forming population, MUC5B, (MG1) and the lower molecular weight (M_r _1.2–1.5 × 10^5 ^Da) non-polymerizing population MUC7 (formerly known as MG2) [3-6]. MUC7 is mainly found in the sol-phase of saliva and is much less abundant in the gel-phase. MUC7 is not a structural component of the acquired pellicle formed on dental and mucosal surfaces around the mouth tissues [7-9]. The glycosylation pattern of these two mucins is also essentially different. MUC7 displays a relatively simple and a unique *O*-linked oligosaccharide profile that is consistent between individuals. In contrast, MUC5B has a much more complex *O-*glycan profile showing substantial inter-individual variations [10].

One of the major functions of MUC7 is to competitively bind to the bacteria in soluble phase of saliva in order to protect potential attachment sites on the tooth and mucosal surfaces from bacterial binding. Considerable data suggest that MUC7 is the primary salivary mucin that binds to oral pathogens [11-14] and also that MUC7 interacts with other protective salivary components including lactoferrin [15] and secretory Ig A [16].

Streptococci, including *S. gordonii*, are the primary colonizers of the dental and mucosal surfaces of the oral cavity and the major constituents of dental plaque [17,18]. They are also common aetiological agents of infective endocarditis [19]. Binding of the bacteria to the acquired pellicle is one of the first steps in the formation of dental plaque. The bacteria can also bind to the pre-formed bacterial layer (coaggregation). Bacterial adherence to these different surfaces is achieved by cell surface proteins, termed adhesins. Substrates may be host derived molecules and other cells. A number of distinct families of streptococcal adhesins are found and characterized based on the molecular organization such as cell wall anchored adhesins [20,21], lipoprotein adhesins [22,23], and anchorless adhesins [24]. The adhesion process is accomplished by protein (lectin)-carbohydrate and/or protein-protein interactions [25].

There is growing interest in the interaction between MUC7 and streptococci. There are reports that MUC7 can interact with various strains of streptococci [26-30], however, reports that identify the specific cell surface proteins/adhesins are rather limited. The purpose of the current study was to identify and characterize the surface proteins involved in the binding of *Streptococcus gordonii *to salivary mucin MUC7. Here we show that human saliva derived MUC7 binds at least four proteins, indicating a complex interaction and further highlights the role of MUC7 in oral mucosal innate defense.

## Methods

**Isolation of MUC7 **was carried out according to a previously described method [31], which employed a two-step chromatographic protocol. Saliva, from a healthy male donor, was collected into an equal volume of 8 M GuHCl, then chromatographed on a column of Sepharose CL-4B eluted with 4 M GuHCl. MUC7-containing fractions, as assessed by immunoblotting, were pooled and chromatographed on a Pharmacia Mono Q HR 10/10 column, eluted with a linear gradient of 0–0.4 M lithium perchlorate/6 M urea/10 mM piperazine, pH 5, as previously described [32]. Fractions showing MUC7-immunoreactivity were pooled then dialyzed gradually against phosphate buffered saline (PBS).

### Streptococcal strains and culture conditions

The PK488 strain of *Streptococcus gordonii *was supplied by Dr. A.J.Jacob (University of Manchester). The strain is identical to ATCC 51656 (American Type Culture Collection, Manassas, VA, USA) [33]. The bacteria was maintained on brain heart infusion agar plates containing 0.5% glucose at 4°C. The strain was subcultured onto the medium every two weeks. Batch cultures of the organism were grown at 37°C to late log phase (16–18 h) in brain heart infusion medium with 5% CO_2 _support.

### Extraction of streptococcal cell surface proteins of the Streptococci

The bacteria were harvested by centrifugation for 10 min at 4,000 g 10°C, then subsequently washed three times in PBS. Bacterial suspensions were then adjusted to an OD at 600 nm = 0.8 with PBS and washed twice with PBS. After each wash the bacteria were pelleted by centrifugation. Finally, the Streptococcal pellet was re-suspended in PBS containing 2% (w/v) SDS, vortexed and incubated at room temperature for 1 h. Next, the SDS-extract was centrifuged at 10,000 rpm at 4°C for 10 min and the supernatant containing surface extract was stored at -80°C for further use. Protein content of the extracts was measured by BCA protein assay kit (Pierce Chester, UK).

### Analytical SDS-PAGE

Sodium dodecyl sulfate-polyacrylamide gel electrophoresis (SDS-PAGE) was performed in a LKB 2050 mini-gel electrophoresis unit in a discontinuous gel system under non-reducing conditions. Samples were mixed with loading buffer [1.25 M Tris-HCl/10% SDS (w/v)/50% (v/v) glycerol containing 0.02% (w/v) bromophenol blue]. Gels were electrophoresed (running buffer; 0.2 M Glycine/0.25 M Tris-HCl, pH 8.3 containing 0.1% (w/v) SDS) at 120 V until the dye front reached the end of the gel. Prestained broad range molecular weight markers were run on every gel. Following electrophoresis, gels were stained with Brilliant blue G-colloid for 2 h, then destained with repeated rinses of 25% (v/v) methanol. Molecular masses of the proteins were automatically calculated in a Bio-rad model GS-700 imaging densitometer with the Profile analyst II, V. 3.11 software.

### Preparative SDS-PAGE

The streptococcal cell surface extract was fractionated on a Bio-Rad Model 491 Prep Cell. A 5 ml sample containing 20 mg Streptococcal surface protein was loaded on a mini-Prep Cell tube (diameter of 37 mm) prepared with a 9 cm 7.5% separating and 4 cm 4% stacking gel. The sample was electrophoresed at 4°C, at constant 60 mA and the elution buffer (0.2 M Glycine/0.25 M Tris-HCl, pH 8.3 containing 0.1% (w/v) SDS) flow velocity of 125 μl/min. 2.5 ml fractions were collected and stored at -80°C for further use.

### Western transfer of SDS-PAGE gels

Gels were equilibrated in transfer buffer [250 mM Tris/20% (v/v) methanol/200 mM glycine containing 0.1% (w/v) SDS] for 15 min prior to transfer to 0.2 μm pore size nitrocellulose membranes using semidry electrotransfer with a Pharmacia-LKB Multiphore II Novablot unit. Transfer conditions were 60 mA constant for 1 h. Identical blots were stained with amido black (0.2% (w/v), containing 3% (w/v) TCA) and destained with methanol, to check transfer efficiency.

For enolase immunoblotting, the membrane was probed with an antibody raised against human enolase (C-19, Santa Cruz) which was shown to cross-react with streptococcal enolase [34]. Immuno-detection was performed using ECL detection.

### Blot overlay assay to detect MUC7-binding proteins from *S. gordonii*

MUC7-binding proteins were determined by an immunoblotting procedure using the monoclonal antibody AM-3. This antibody is reactive against the oligosaccharide structure sialyl-Lewis^x ^which is present on MUC7 [35,36]. After the western transfer of the Streptococcal surface extract, the membranes were washed in PBS 2 × 5 min and then blocked with TBST (Tris buffered saline-Tween – 10 mM Tris-HCl/150 mM sodium chloride, pH 8.0 containing 0.05% (v/v) Tween 20) supplemented with 1% (w/v) skimmed milk powder 30 min. They were rinsed twice with PBS 10 min, and incubated with MUC7 preparation (10 μg/ml in PBS) at 4°C overnight. In the meantime, a replica membrane was incubated with PBS as control. After the incubation the membranes were rinsed twice for 20 min with TBST. The membranes including replica control, were then incubated with AM-3 in TBST (1:50 dilution) for 1 h, then rinsed with TBST 2 × 10 min and incubated with secondary antibody (IgM anti-mouse, peroxidase conjugated, 1:2000 dilution) in TBST for 30 min. The membranes were rinsed with TBST 3 × 10 min. ECL detection was carried out using an Amersham ECL kit according to the manufacturer's instructions.

### Anti-enolase labelling and flow cytometry analysis of the bacteria

*S. gordonii *suspension was adjusted to OD at 250 nm of 0.5 with PBS and incubated with an anti-enolase antibody (C-19, Santa Cruz) overnight at 4°C with end-over-end rotation. The bacteria were harvested by centrifugation at 3000 × g at 4°C, washed twice with ice-cold PBS. Texas Red-labeled anti-goat IgG (Jackson ImmunoResearch) secondary antibody was added to the bacterial suspension and incubated for 30 min and then washed with PBS as described above. Purified goat IgG (Invitrogen) was incubated with the bacteria and used as isotype-matched control. Samples were analyzed by a CyAn ADP flow cytometer (Beckman Coulter) and the data were analyzed using Summit software version 4.3. A minimum of 2 × 10^4 ^cells per sample were examined.

### In-gel digestion

A previously described method [37] was used for in-gel digestion of the putative adhesins with some minor modifications. Briefly, the protein band was cut out from the SDS-PAGE gel and transferred into a 1.5 ml eppendorf tube; all subsequent steps were performed in the same tube. Gel pieces were de-stained with 50 mM NH_4_HCO_3 _in 50% acetonitrile and then reduced with 10 mM dithiothreitol in 50 mM NH_4_HCO_3 _at 37°C for 1 h prior to alkylation by addition of 55 mM iodoacetamide 1 h in the dark at room temperature. The gel pieces were washed in 100 mM NH_4_HCO_3 _before dehydrating in acetonitrile and then rehydrating in 100 mM NH_4_HCO_3_. Gel pieces were dehydrated once again in acetonitrile and dried in the vacuum centrifuge (about 30 min). Trypsin (1 ng/μl in 50 mM NH_4_HCO_3_) was added to the dried gel pieces and left for 30 min in ice. Excess digestion buffer was replaced with the same buffer (10 μL) without trypsin and the gel pieces were incubated 24 h at 37°C. Extraction of the peptides was performed in two steps; 50 μL of 25 mM NH_4_HCO_3 _for 30 min and 50 μL of 5% (v/v) formic acid in 50% acetonitrile (v/v) 2 × 20 min. Extracts obtained from each step, were combined, then dried down and analyzed by LC MS/MS.

### Protein identification by tandem mass spectrometry

Digested samples were introduced to a Waters Q-Tof *micro*, hybrid quadropole orthogonal acceleration time-flight mass spectrometer via a Waters CapLC system which was configured with a PepMap™ C18 (LC Packing, 300 μm ID × 5 mm) pre-concentration column in series with a Atlantis^® ^(Waters) dC18 NanoEase™ (75 m × 150 mm) nanoscale analytical column. Samples were separated on the column with a gradient of 5% acetonitrile in 0.1% formic acid to 60% acetonitrile in 0.1% formic acid over 45 min. All data were acquired using Masslynx 4.0 software. The mass spectrometer data directed analysis (DDA) acquired MS survey data from m/z 200 to 1500 with the criteria for MS to MS/MS including ion intensity and charge state using a 1-second MS survey scan followed by 1.5-second MS/MS scans, each on three different precursor ions. The Q-Tof micro was programmed to ignore any singly charged species and the collision energy used to perform MS/MS was carried out according to the mass and charge state of the eluting peptide. Precursors detected were excluded from any further MS/MS experiment for 180 seconds. All analyses were repeated twice for each sample, and peptides identified in the first run were excluded from the second analysis.

### Data processing and database searching

The raw data acquired were processed using Proteinlynx module of Masslynx 4.0 to produce *.pkl (peaklist) files. The peptide QA filter was 30 to eliminate poor quality spectra and the minimum peak width at half height was set to 4 to eliminate background noise peaks. Smoothing (x2 Savitzky Golay) and polynomial fitting were performed on all peaks and the centroid taken at 80% of the peak height. The data processed were searched against National Center for Biotechnology Information (NCBI) non-redundant (nr) protein database (version 20050805; 2,739,666 sequences) and Swiss-Prot (Release 48.7; 190,255 sequences) using an in house MASCOT (Matrix Science, UK) search engine (Version 2.0). Parameters used for the MASCOT search were: Taxonomy Bacteria (Eubacteria), 0.2 Da mass accuracy for parent ions and 0.3 Da accuracy for fragment ions, one missed cleavage was allowed, carbamidomethyl-modification of cysteine and methionine oxidation were used as fixed and variable modifications respectively.

## Results

### Purification of MUC7

**Figure 1:**
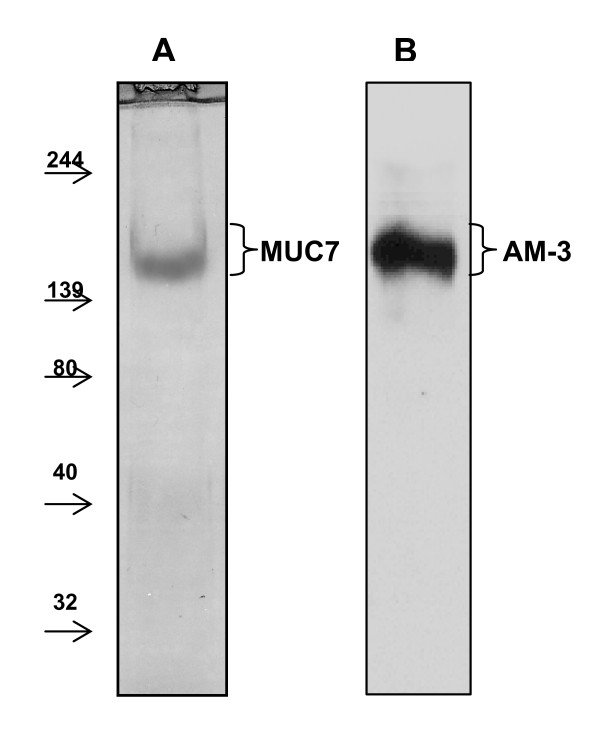
**SDS-PAGE and Western blot analysis of purified MUC7 preparation**. MUC7 purified by employing a two-step chromatographic protocol as described in Methods. **(A) **Final purified MUC7 pool from Mono Q HR 10/10 ion exchange column was electrophoresed in a Midget 7.5% SDS-PAGE gel under reducing conditions and visualized by Coomassie blue staining and Western transferred to nitrocellulose membranes and probed with AM-3 monoclonal antibody **(B)**. Positions of the molecular weight markers are indicated (kDa).

A rapid two step chromatographic protocol as described by Mehrotra et al. [31] was applied to purify MUC7 from the saliva. This method provided the recovery of this molecule at high purity and in adequate amount (750 μg/ml, as assessed by refractive index measurement, data not shown), enabling MUC7-streptococcus binding studies. Purity of the MUC7 preparation was assessed by SDS-PAGE, Western blotting and mass spectrometry. The final purified MUC7 pool from the Mono Q HR 10/10 ion exchange column was electrophoresed in a Midget 7.5% SDS-PAGE gel under reducing conditions and visualized by Coomassie blue staining (Figure 1A). The pool contained a detectable amount of a protein with apparent M_r _170 kDa, while no other proteins were visualized. This protein band was subjected to in-gel digestion and the resultant peptides were analysed by LC-MS/MS. Three peptides (**SHFELPHYPGLLAHQKPFIR**, **LPPSPNNPPK**, and **FLLYMK**) from the MUC7 core protein were clearly identified by mass spectrometry. The gel was also transferred to nitrocellulose membranes and probed with the AM-3 monoclonal antibody. AM-3 reactivity showed one distinct band at the same region with Coomassie blue stained protein which was later identified as MUC7 (Figure 1B).

### Extraction and separation of SDS-extracted Streptococcal surface proteins

**Figure 2:**
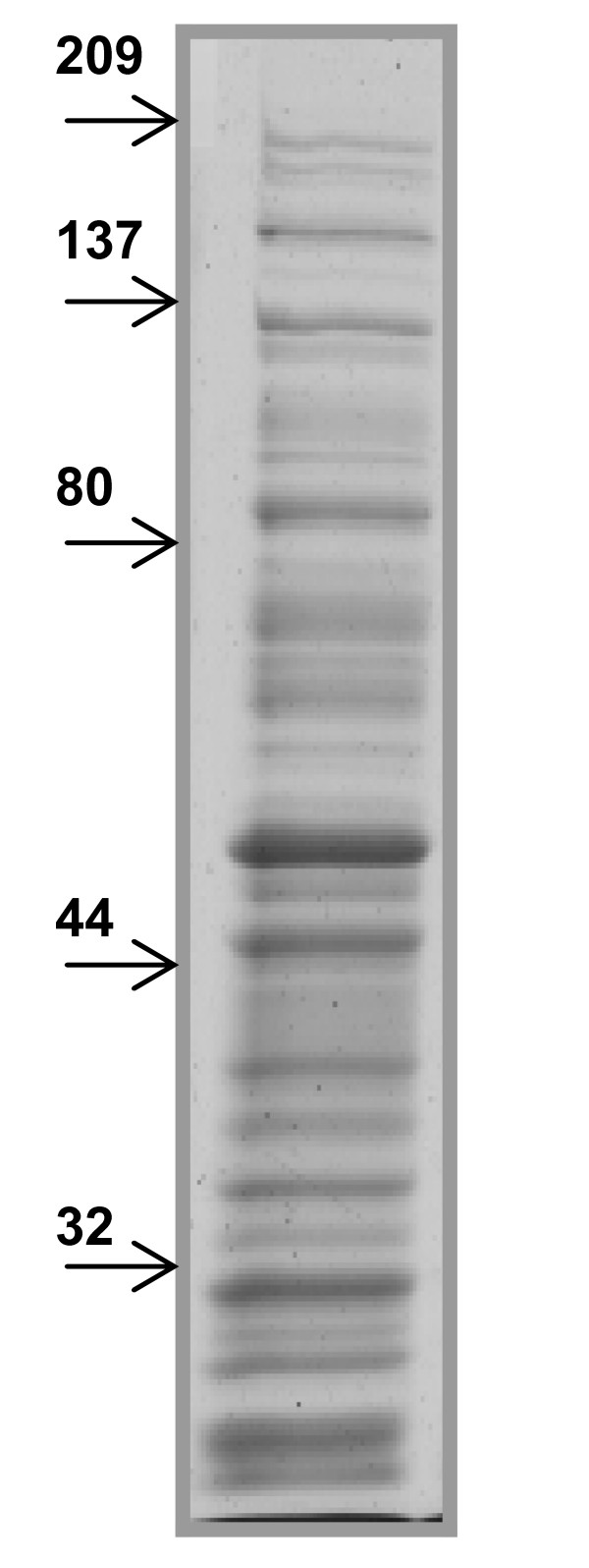
**Protein profile of SDS-extracted surface proteins from *S. gordonii***: 10 μg of the SDS-extract supernatant from S. gordonii was electrophoresed on a 10% SDS-PAGE gel under non-reducing condition. Separated proteins were stained by Coomassie blue. Positions of the molecular weight markers are indicated (kDa). Results are shown as one representative experiment of three different *S. gordonii *preparations.

SDS-extracted proteins from intact *S. gordonii *were separated by SDS-PAGE under non-reducing conditions (Figure 2). The extract yielded a large number of bands; at least 30 bands were observed on the gel. In order to check for possible cell lysis and hence contamination by intracellular proteins, the extract was examined for presence of DNA by UV spectrophotometry but none was detected (260/280 ratio was smaller than 0.6, data not shown).

### Identification of Putative MUC7 binding proteins by blot overlay assay

**Figure 3:**
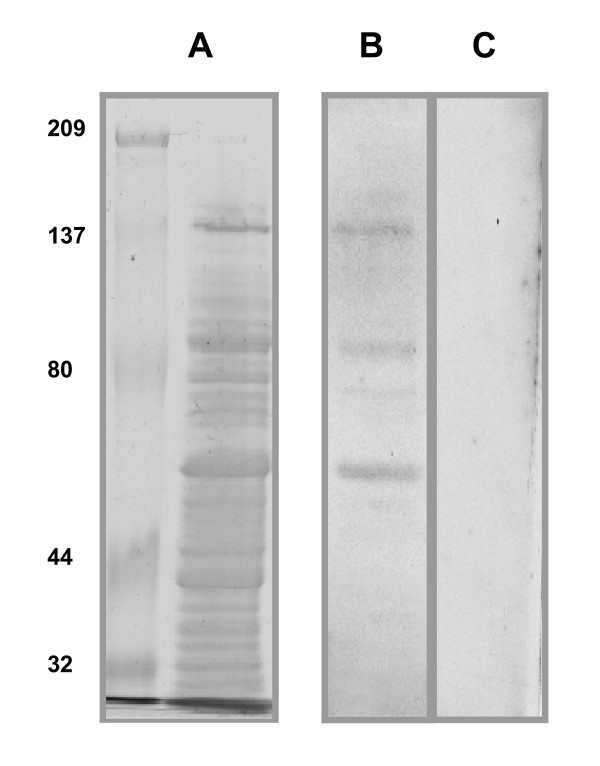
**Identification of putative mucin binding proteins by blot overlay assay**. SDS-extracted putative surface proteins were separated by a 7% SDS-PAGE and Western blotted onto nitrocellulose and incubated with purified MUC7 preparation (50 μg/ml). Binding of MUC7 to putative surface proteins determined by immunological procedures probing the membrane with AM-3 antibody and ECL detection **(B)**. Molecular masses of the MUC7-binding proteins were calculated in Bio-rad model GS-700 imaging densitometer and it's PC compatible software. A control Western blot, which had been incubated with PBS instead of MUC7 preparation was probed with AM-3 antibody and subjected to ECL detection **(C)**. The efficiency of the Western transfer of the separated SDS-extracted proteins was assessed by amido black staining of the membranes **(A)**. Positions of the molecular weight markers are indicated (kDa). Results are shown as one representative experiment of multiple independent preparations.

In order to identify streptococcal proteins that bind MUC7, the SDS-extracted proteins were Western blotted onto nitrocellulose membranes and incubated with the MUC7 preparation. Mucin binding was quantified by immunoblotting with an antibody against a glycan on MUC7. The transfer of the separated proteins to nitrocellulose membranes was assessed by a visual comparison of blots stained with amido black compared to replica SDS-PAGE gels stained with Coomassie blue (Figure 3A). The comparison shows that all bands seen in the SDS-PAGE gel (Figure 2) were represented on the membrane. The extracted and separated proteins were blotted onto nitrocellulose and subsequently incubated with purified MUC7 (50 μg/ml) preparation. Detection of bound MUC7 with monoclonal antibody AM-3 identified several putative adhesin bands with apparent molecular mass **62, 78, 84, 133 **kDa (Figure 3B). A control replica Western blot probed with monoclonal antibody AM-3 and secondary antibody without prior incubation with MUC7 did not visualize any bands (Figure 3C).

**Figure 4:**
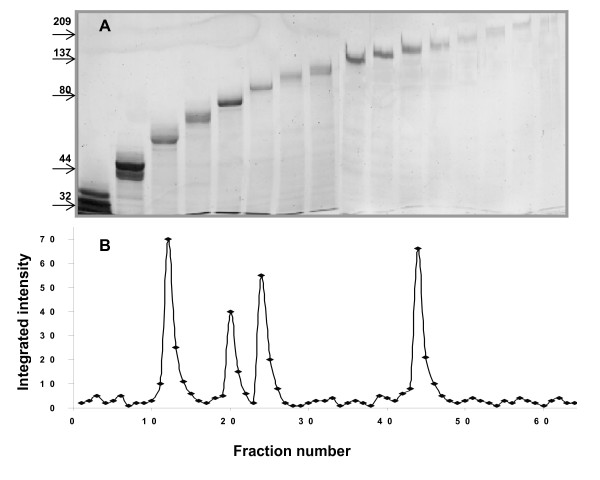
**Preparative SDS-PAGE of SDS-extract from Streptococcus gordonii PK488 and identification of MUC7 binding proteins**. Twenty milligrams of the surface extract from *S. gordonii *was electrophoresed on a 7.5% preparative electrophoresis in a Bio-Rad mini-prep cell and **(A) **selected fractions were electrophoresed on 7.5% SDS-PAGE gels, proteins visualized with silver stain. **(B) **Selected fractions were transferred onto nitrocellulose membranes by slot blotting and probed with MUC7 preparation. MUC7 binding was determined by immunoblotting as described in Material and Methods. Positions of molecular weight markers are indicated (kDa).

Further characterization of the MUC7-binding proteins required their preparative separation and purification; hence, the SDS-extracted proteins from intact *S. gordonii *were fractionated by preparative SDS-PAGE and the resulting fractions were analyzed by analytical SDS-PAGE (Figure 4). The electrophoretic analysis of the selected fractions indicated that putative MUC7-binding bands could be separated from other streptococcal proteins (Figure 4A). This separation of the adhesin bands from the nearest contaminant allowed a cleaner sample for in-gel digestion and subsequent protein identification. In order to determine the fractions that contained MUC7 binding proteins, aliquots of the fractions from the preparative electrophoresis were transferred to the nitrocellulose membranes by slot blotting and probed with 50 μg/ml MUC7 in PBS (Figure 4B). Antibody reactivity was detected around the fractions 12–13 (62 kDa), 20–21 (74 kDa), 24–25 (84 kDa) and 44–45 (133 kDa), confirming the result obtained from Western transfer and following overlay assay as described above.

Putative adhesin bands were subjected to in-gel digestion and the resultant peptides were analyzed by LC-MS/MS. Database searching using the MS/MS peptide fragmentation data revealed identification of each band with high probability identity scores and extensive homology (p < 0.005) (table 1). Two significant protein identifications were revealed from the 133 kDa band: one was streptococcal ***Enolase ***(15 peptides, 37% coverage, Mr 47 kDa) and the other was streptococcal ***DNA-directed RNA polymerase, beta' subunit ***(11 peptides, 13% coverage, Mr 135 kDa). The 84 kDa band also contained two streptococcal proteins; ***translation elongation factor G, EF-G ***(47 peptides, 53% coverage, Mr 76 kDa), and ***SecA ***protein (7 peptides, 10% coverage, Mr 95 kDa). The 78 kDa band was identified as ***oligopeptide-binding lipoprotein ***(4 peptides, 6% coverage, Mr 74 kDa). ***Translational elongation factor, EF-Tu ***(57 peptides, 55% coverage, Mr 43,943), was the major protein in the 62 kDa band.

**Table 1:** Identified proteins by LC-MS/MS analysis from the digestion of putative adhesin bands. Proteins are ranked according to their probability score.

Gel digestion	Protein hits	Species	Mw	Score/peptides/coverage
133 kDa band*	**1- alpha Enolase**	*S. gordonii*	47,103	**727/15/37%**
	2- DNA-directed RNA polymerase, beta' subunit	Streptococcus	134,965	560/13/13%

84 kDa band*	**1- translation elongation factor G, EF-G**	Streptococcus	76,620	**1251/47/53%**
	2- SecA	*S. gordonii*	95,193	229/7/10%

78 kDa band*	**1-Oligopeptide-binding lipoprotein**	*S. gordonii*	76,015	**438/12/18%**
	2- Heat shock protein, chaperonin	*S. termophilus*	64,738	197/4/6%

62 kDa band*	**1-Translation elongation factor Tu, EF-Tu**	Streptococcus	43,943	**1135/57/55%**
	2- Pyruvate kinase	Streptococcus	54,777	467/9/24%

* Molecular masses of the putative adhesin bands were calculated in Bio-rad model GS-700 imaging densitometer and it's PC compatible software.

**Figure 5:**
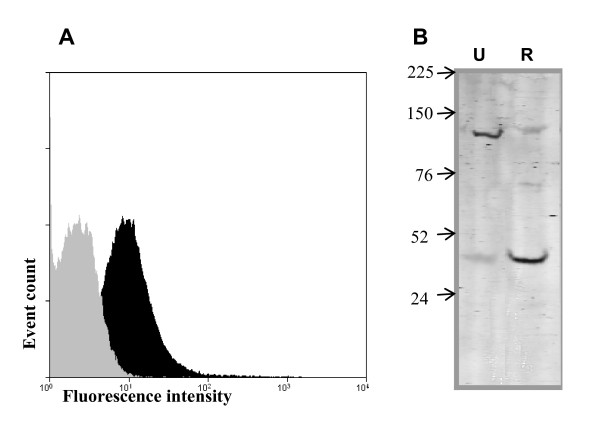
**Flow cytometry and SDS-PAGE analysis of *S. gordonii *surface enolase**. **A)**- Intact *S. gordonii *preparation was stained with a polyclonal antibody for α-enolase (C-19). Specific secondary antibody coupled with Texas Red (anti goat) was used for detection (filled black) and compared with isotype control (filled gray). Results are shown as one representative experiment of three different *S. gordonii *preparations. **B)**- An aliquot from the surface extract from *S. gordonii *were separated on a 4–20% gradient SDS-PAGE gel, unreduced **(U, lane 1) **and reduced **(R, lane 2)**. The gel was Western blotted onto nitrocellulose membrane and probed with anti-enolase antibody. Positions of the molecular markers are indicated (kDa).

The majority of the putative MUC7-binding proteins identified are supposedly intracellular proteins suggesting the SDS-extraction had caused cell lysis. To address this issue, we performed flow cytometry analysis using an anti-α-enolase antibody to investigate whether this protein was present at the cell surface of *S. gordonii*. The bacteria showed a strong signal for α-enolase indicating its cell surface expression (Figure 5a). It is noteworthy that α-enolase which has a predicted Mr of 47 kDa was observed to have an apparent Mr of 133 kDa (table 1 and Figure 5B–U). However, boiling with SDS and/or reduction of the extract resulted in a change in apparent Mr to the expected value of approx. 47 kDa (Figure 5B–R).

## Discussion

MUC7 is responsible for modulation of the oral microbial flora by selective attachment and following clearance of certain microorganisms. There are some reports that MUC7 can adhere to various strains of streptococci [26-30] which are the primary colonizers and predominant microorganisms of the oral cavity. In order to further understand these interactions and their consequences, the specific streptococcal surface proteins, in other word adhesins, that bind MUC7 must be identified. Although there has been growing interest in MUC7-streptococcal interaction, there are limited reports that have identified specific MUC7 binding adhesins in the literature. Here we have identified, using highly purified MUC7 mucin in a blot overlay assay of SDS extracted *S. gordonii *proteins, a number of putative MUC7-specific binding proteins.

At first glance, the majority of the proteins identified as putative MUC7 binding proteins appear to be intracellular in origin, however, there are growing reports in the literature that most of these proteins can also be present on the surface of the bacteria and are involved in extracellular interactions (see below). Although these proteins do not have a signal sequence, they are somehow secreted by an unknown mechanism and are believed to associate with the bacterial surface to become functional [24].

Tandem mass spectrometry analysis of the 133 kDa band identified the glycolytic enzyme enolase and the β-subunit DNA-directed RNA polymerase, both supposedly intracellular proteins. However, presence of cell surface enolase and its interaction with extracellular plasmin(ogen) has been shown in a number of studies on different streptococcal species [38-41]. It has also been shown that surface α-enolase from *Streptococcus mutans *interacts with human plasminogen and salivary mucin MG2 (MUC7) [26]. Indeed, we provide evidence here by flow cytometric analysis that α-enolase is present at the surface of *S. gordonii*. It is noteworthy that the 47 kDa enolase protein was identified from the digestion of 133 kDa band, suggesting its possible oligomerization and/or modification, perhaps glycosylation or interaction with other proteins. Our immunoblot analysis, using an α-enolase antibody indicated that boiling with SDS and/or using a reducing agent moves the anti-enolase response from 133 kDa to the 47 kDa region (Figure 5B) suggesting an interaction with itself or other protein(s). The other protein identified in the 133 kDa band was DNA-directed RNA polymerase (RNAP) which is mainly located in the cytoplasm, however, Beckman and coworkers [42], demonstrated that DNA-directed RNA polymerase subunit from Group B streptococci is a candidate cell surface protein that binds to the extracellular matrix protein, fibronectin.

We have also identified elongation factors as putative MUC7-binding proteins; the 84 kDa and 62 kDa protein bands were shown to contain EF-G and EF-Tu respectively. This is again somewhat surprising since EF-Tu, in general, is an intracellular protein that promotes the GTP-dependent binding of aminoacyl-tRNA to the a-site of ribosomes during protein biosynthesis [43]. However, there are several reports that some intracellular proteins, including elongation factors EF-G, EF-Ts, EF-P, and EF-Tu, can be localized on the cell surface of the pathogens and interact with extracellular proteins [39,41,44,45]. Furthermore, it has been demonstrated in a previous study that elongation factor Tu (Ef-Tu) from *Lactobacillus johnsonii *is the main cell surface protein that mediates its binding to intestinal epithelial cells and mucins [46].

Expression of cell surface lipoproteins of *Streptococcus gordonii *is related to its adherence and coaggregation [22]. It has been shown previously that the 76 kDa lipoprotein, termed SarA (*hppA*) from *S. gordonii *is a crucial cell surface protein that enables the bacteria to aggregate and coaggregate with certain microorganisms [23]. Here, we have clearly identified that the 78 kDa putative MUC7-binding band contains the *hppA *gene product, oligopeptide binding lipoprotein. This cell surface lipoprotein has been shown to be essential for uptake of hexa- and heptapeptides as source of nutrients to the organism [47]. Our results indicate that MUC7 binds to this lipoprotein adhesin; possibly this binding hinders the lipoproteins function in nutrient uptake and preventing adhesion and aggregation to the mucosal and/or dental surfaces.

Detergent extraction of surface proteins from different streptococcal species has been successfully applied to study different aspects of their surface proteins, including identifying mucin binding adhesins [48,49]. In the current study, extraction of streptococcal cell surface proteins was achieved by SDS, which has been used previously to extract lipoprotein adhesins from *S. gordonii *[47,50]. The SDS-PAGE profiles of the SDS extracted proteins observed here are in general agreement with published data [51].

In order to identify MUC7 binding proteins from *S. gordonii*, a blot overlay assay was employed. This method has been successfully employed to investigate mucin-bacteria interactions by various investigators [22,44,46]. For example, Murray *et al*. [52] demonstrated that detergent-extracted *S. gordonii *surface proteins were able to bind a trisaccharide that is later shown as a major oligosaccharide structure on MUC7 [53]. Furthermore, Carnoy *et al*. [54] used a similar strategy that was employed here (western blotting of extracted bacterial protein and subsequent probing with mucins) to identify *Pseudomonas aeruginosa *outer membrane adhesins that bind respiratory mucins. However, none of these studies have identified the specific bacterial proteins that bind to the mucins.

## Conclusion

In summary, the identification and characterization of specific mucin binding proteins is crucial to understand host-pathogen interaction. Here we have identified putative MUC7-binding surface proteins from *Streptococcus gordonii*. Additional experiments should be done to confirm and further characterize the interaction of these proteins with the mucin and their *in vivo *significance. Moreover, their role with respect to bacterial pathogenesis and host defense remains to be elucidated.

## Abbreviations

MUC7: mucin 7; MG2: mucus glycoprotein2; MS: mass spectrometry, EF-Tu: elongation factor Tu;

## Competing interests

The authors declare that they have no competing interests.

## Authors' contributions

MK had the primary responsibility of the planning of the study and carried out the experiments, RM and DJT contributed to the planning of the study and experiments, NK and JKS directed the design and execution of the project. All authors have read and approved the manuscript.
